# Characteristics of lung metastasis in testicular cancer: A large-scale population analysis based on propensity score matching

**DOI:** 10.3389/fsurg.2022.959573

**Published:** 2022-11-04

**Authors:** Aiyuan Guo, Jie Gu, Jiayi Yang

**Affiliations:** ^1^ Department of Dermatology, The Third Xiangya Hospital, Central South University, Changsha, China; ^2^ Department of Geriatric Urology, Xiangya International Medical Center, Xiangya Hospital, Central South University, Changsha, China; ^3^ National Clinical Research Center for Geriatric Disorders, Xiangya Hospital, Central South University, Changsha, China; ^4^ Department of Geriatrics, Xiangya Hospital, Central South University, Changsha, Hunan, China

**Keywords:** testicular cancer, lung metastasis, propensity score matching, nomogram, risk factors, survival analysis

## Abstract

**Background:**

This study aims to systematically evaluate predictive factors for lung metastasis (LM) in patients with testicular cancer (TC) and to investigate cancer-specific survival (CSS) and overall survival (OS) of LM in TC patients based on a large population-cohort.

**Methods:**

A total of 10,414 patients diagnosed with TC during 2010–2015 were adopted from the Surveillance, Epidemiology, and End Results (SEER). After propensity score matching (PSM), 493 patients with LM were included for subsequent analysis. Univariate and multivariate logistic regression analyses were employed to identify risk factors, a nomogram was developed, and the receiver operating characteristic (ROC) curve was utilized to confirm the validation of the nomogram. Prognostic factors for OS and CSS among TC patients with LM were estimated *via* Cox proportional hazards models.

**Results:**

Postmatching indicated that 11 parameters were successfully balanced between both groups (*P* > 0.05). After PSM, TC patients with LM presented an undesirable prognosis in both CSS and OS than those without LM (*P* < 0.001). The logistic regression model showed that tumor size; T stage; N stage; liver, brain, and bone metastases; and histology were positively associated with LM (*P* < 0.05). A nomogram was developed to predict diagnostic possibilities based on the independent risk variables, and the ROC curve verified the predictive capacity of the logistic regression model [area under the curve (AUC) = 0.910].

**Conclusion:**

The selected variates in the nomogram can be predictive criteria for TC patients with LM. Brain metastasis, liver metastasis, and larger tumor size were prognostic factors for CCS and OS among TC patients with LM.

## Introduction

Testicular cancer (TC) is the most common malignancy among young men, gradually increasing over recent decades (1). Nonseminomatous germ cell tumor (NSGCT) is one of the major pathological types of TC, in which about half of the patients present with metastatic disease (2). For metastatic TC, the combination treatment is closely related to improved survival. However, TC is the only tumor with a mean age of survivors younger than 65 years, and its 5-year survival decreases sharply from 99% (without metastases) to 74% (metastases) (3, 4). In this regard, characterizing metastatic TC risk factors based on variant clinical outcomes is indispensable.

The most common metastatic site in TC patients is the lungs (5, 6). The incidence of TC metastasizing to the lungs has been increasing in recent years (3), possibly due to the improved diagnostic tools and appliance of developed multidisciplinary management on metastatic lung lesions such as surgical removal, radiation, salvage treatments, and systemic chemotherapy, which may contribute to the increased survival time and incidence of lung metastasis (LM) in TC patients (7–10). Furthermore, the treatments of metastatic TC have a profound impact on the quality of life of patients, such as sexual function, reproductive capacity, and psychological situation (11, 12). Unfortunately, risk factors and population-level estimates for prognosis related to the development of LM among TC patients, which removed confounding factors, have not been extensively studied.

Therefore, we employed propensity score matching (PSM) for the first time to balance the confounding biases between testicular patients with and without LM. What's more, we developed a nomogram to predict the probability of LM in TC patients.

This study is based on the Surveillance, Epidemiology, and End Results (SEER) database to identify predictive factors for LM in TC patients. Due to the poor prognosis of LM in TC patients and because its etiology remains unclear, prognosis factors such as clinical and sociodemographic predictors of poor survival in these patients were also investigated to assess the overall and cancer-specific survival (CSS) of TC patients with LM.

## Methods

### Study population

TC patients were identified from the SEER database (13). Since the details of LM were not available before 2010, primary TC patients initially diagnosed between 2010 and 2015 were collected (the latest data is on December 31, 2015). Patients diagnosed with TC from January 1, 2010 to December 31, 2015, were adopted to analyze risk factors.

### Statistical analysis

Patients' demographic and clinical characteristics are presented as follows: age (<50 and ≥50 years), race (White, Black, others), marital status (married or unmarried), tumor grade (I = well-differentiated, II = moderately differentiated, III = poorly differentiated, IV = undifferentiated and anaplastic, unknown), primary tumor stage (T stage: T1, T2, T3, and T4), regional lymph node stage (N stage: N0, N1, N2, and N3), primary site (testis, descended testis, and undescended testis), histology (seminoma, nonseminoma, nongerminal neoplasm, and mixed tumor), laterality [left, right, bilateral, only one side (unspecified), and paired site], the presence of bone metastasis, liver metastasis, brain metastasis, tumor size (<67 mm and ≥67 mm), lymph nodes (regional, distant, none, and unknown), and lymph nodes size (≤2 cm, >2 cm and ≤5 cm, >5 cm, none). The differences in the LM incidence between the categorical variables were analyzed by Pearson's *χ*^2^ test or the rank-sum test.

PSM analysis (nearest-neighbor matching) was then utilized to adjust for differences between LM and non-LM TC patients (14). Propensity scores were analyzed using R (version 3.5.1). The covariates used in generating the propensity score included age, race, marital status, grade, primary site, histology, laterality, bone metastasis, liver metastasis, brain metastasis, T stage, N stage, tumor size, lymph nodes, and lymph node size. After PSM, 493 patients with LM were matched with patients without LM at a 1 : 1 ratio to assess the difference in survival probability (OS and CSS).

The risk factors for TC patients with *de novo* LM were determined by univariate and multivariate logistic regression analyses. The logistic regression model, which adopted the bidirectional elimination method and was optimized by the Akaike information criterion (AIC) protocol, was used to screen the risk factors. Then, a nomogram was developed by using the RMS package in R version 3.5.1 (http://www.r-project.org/). Harrell's *C* index and receiver operating characteristic (ROC) curve were used to evaluate the validation of the nomogram, which could assess the consistency between the actual and predicted results. The calibration curve described the average predictive estimate against actual observation and evaluated the nomogram performance visually, and the calibration plot was used for internal validation.

Prognostic factors for OS and CSS among TC patients with LM were estimated using univariate and multivariate analyses with Cox proportional hazards models. All statistical analyses were performed using R version 3.5.1; *P*-value < 0.05 was considered statistically significant.

## Results

### Demographic and clinical characteristics

A total of 10,414 patients diagnosed with TC during 2010–2015 met the inclusion criteria. The mean age of the patients at diagnosis was approximately 34.31 years, most of whom were White (91.16%). Among these patients, 493 patients were diagnosed with TC with LM during 2010–2015. The demographic and clinical characteristics of the included patients are presented in Table 1.

**Table 1:** Baseline of the demographic and clinical characteristics for patients diagnosed with testicular cancer.

Subjects’ characteristics	No. of testicular cancer patients		*χ* ^2^	*P-*value
	With lung metastasis	Without lung metastasis		
	(*N* = 493, 4.73%)	(*N* = 9,921, 95.27%)		
Age (years)			7.944	0.005
<50	457 (92.7)	8,777 (88.5)		
≥50	36 (7.3)	1,144 (11.5)		
Race			0.140	0.933
Black	16 (3.2)	293 (3.0)		
White	448 (90.9)	9,045 (91.2)		
Others	29 (5.9)	583 (5.9)		
Marital status			75.395	<0.001
Yes	119 (24.1)	4,374 (44.1)		
No	374 (75.9)	5,547 (55.9)		
Grade			21.175	<0.001
I	0 (0.0)	45 (0.5)		
II	1 (0.2)	14 (0.1)		
III	10 (2.0)	53 (0.5)		
IV	4 (0.8)	44 (0.4)		
Unknown	478 (97.0)	9,765 (98.4)		
Primary site			3.193	0.203
Testis	235 (47.7)	5037 (50.8)		
Descended testis	253 (51.3)	4,727 (47.6)		
Undescended testis	5 (1.0)	157 (1.6)		
Histology			542.290	<0.001
Seminoma	35 (7.1)	5,775 (58.2)		
Nonseminoma	126 (25.6)	1,177 (11.9)		
Nongerminal neoplasm	23 (4.7)	71 (0.7)		
Mixed tumor	309 (62.7)	2,898 (29.2)		
Laterality			9.341	0.053
Left	232 (47.1)	4,691 (47.3)		
Right	260 (52.7)	5,224 (52.7)		
Bilateral	0 (0.0)	2 (0.00)		
Only one side (unspecified)	1 (0.2)	1 (0.00)		
Paired site (no laterality)	0 (0.0)	3 (0.00)		
Bone metastasis			216.890	<0.001
Yes	20 (4.1)	13 (0.1)		
No	473 (95.9)	9,908 (99.9)		
Liver metastasis			1,076.500	<0.001
Yes	73 (14.8)	23 (0.2)		
No	420 (85.2)	9,898 (99.8)		
Brain metastasis			688.490	<0.001
Yes	39 (7.9)	4 (0.0)		
No	454 (92.1)	9,917 (0.0)		
T stage			662.970	<0.001
T1	169 (34.3)	6,842 (69.0)		
T2	194 (39.4)	2,730 (27.5)		
T3	124 (25.2)	309 (3.1)		
T4	6 (1.2)	40 (0.4)		
N stage			743.410	<0.001
N0	198 (40.2)	8,460 (85.3)		
N1	68 (13.8)	496 (5.0)		
N2	113 (22.9)	566 (5.7)		
N3	114 (23.1)	399 (4.0)		
Tumor size (mm)			88.015	<0.001
<67	326 (66.1)	8,219 (82.8)		
≥67	167 (33.9)	1,702 (17.2)		
Lymph nodes			690.850	<0.001
Regional	3 (0.6)	32 (0.3)		
Distant	253 (51.3)	1,270 (12.8)		
None	198 (40.2)	8,460 (85.3)		
Unknown	39 (7.9)	159 (1.6)		
Lymph node size (cm)			755.550	<0.001
≤2	67 (13.6)	517 (5.2)		
>2 and ≤5	113 (22.9)	548 (5.5)		
>5	115 (23.3)	396 (4.0)		
None	198 (40.2)	8,460 (85.3)		
Overall status			610.180	<0.001
Alive	382 (77.5)	9,696 (97.7)		
Dead	111 (22.5)	225 (2.3)		
Cancer status			826.150	<0.001
Alive	395 (80.1)	9,809 (98.9)		
Dead	98 (19.9)	112 (1.1)		

### Incidence of lung metastasis

A total of 493 patients with TC were diagnosed with LM (4.73%) among 10,414 patients, and the incidences of LM in the testis, descended testis, and undescended testis were 47.7%, 51.3%, and 1.0%, respectively, without a significant difference (*P* = 0.203) (Table 1). Subgroup analysis also showed that younger patients (<50 years) presented a significantly higher incidence of LM than those older patients (≥50 years) (*χ*^2^ = 7.944, *P* = 0.005). Moreover, patients with negative marital status (including single, unmarried, divorced, separated, and widowed patients) (*χ*^2^ = 75.395, *P* < 0.001), mixed tumor histology (*χ*^2^ = 542.290, *P* < 0.001), bone metastasis (*χ*^2^ = 216.890, *P* < 0.001), liver metastasis (*χ*^2^ = 1076.500, *P* < 0.001), brain metastasis (*χ*^2^ = 688.490, *P* < 0.001), higher T stage (*χ*^2^ = 662.970, *P* < 0.001), higher N stage (*χ*^2^ = 743.410, *P* < 0.001), lymph nodes metastasis (*χ*^2^ = 690.850, *P* < 0.001), lymph node size (*χ*^2^ = 755.550, *P* < 0.001), with alive cancer status (*χ*^2^ = 826.150, *P* < 0.001) and alive overall status (*χ*^2^ = 610.180, *P* < 0.001) presented higher LM incidence than their counterparts (Table 1).

### Propensity score matching and survival analysis

To account for confounding bias inherent to TC patients with LM, PSM was used to adjust for differences in all variates (including age, race, marital status, grade, primary site, grade, histology, laterality, bone metastasis, brain metastasis, liver metastasis, T stage, N stage, tumor size, lymph nodes metastases, and lymph node size) (Table 2). Of the 493 available cases with LM, 493 cases without LM were able to be matched, and the matching ratio was 1 : 1. All 15 variables were involved in PSM. However, postmatching *P*-value indicated that 11 variables were successfully balanced between both groups while the other 4 variables (laterality, bone metastasis, brain metastasis, and liver metastasis) failed to be balanced, among which the *P*-value for laterality was NA and the *P*-values for the other three were >0.05.

**Table 2:** Clinical characteristics of testicular cancer patients with lung metastasis after propensity score matching (*n* = 493).

Subjects’ characteristics	No. of testicular cancer patients with LM after PSM (2010–2015)		*χ* ^2^	*P*-value
	With LM (%)	Without LM (%)		
Age (years)			0.14	0.706
<50	457 (92.7)	461 (93.5)		
≥50	36 (7.3)	32 (6.5)		
Race			0.12	0.941
Black	16 (3.2)	18 (3.7)		
White	448 (90.9)	446 (90.5)		
Others	29 (5.9)	29 (5.9)		
Marital status			0.27	0.606
Yes	119 (24.1)	127 (25.8)		
No	374 (75.9)	366 (74.2)		
Grade			NA	NA
I	0 (0.0)	0 (0.0)		
II	1 (0.2)	2 (0.4)		
III	10 (2.0)	9 (1.8)		
IV	4 (0.8)	6 (1.2)		
Unknown	478 (97.0)	476 (96.6)		
Primary site			0.65	0.723
Testis	235 (47.7)	230 (46.7)		
Descended testis	253 (51.3)	260 (52.7)		
Undescended testis	5 (1.0)	3 (0.6)		
Histology			1.52	0.678
Seminoma	35 (7.1)	31 (6.3)		
Nonseminoma	126 (25.6)	122 (24.7)		
Nongerminal neoplasm	23 (4.7)	17 (3.4)		
Mixed tumor	309 (62.7)	323 (65.5)		
Laterality			NA	NA
Left	232 (47.1)	235 (47.7)		
Right	260 (52.7)	258 (52.3)		
Bilateral	0 (0.0)	0 (0.0)		
Only one side (unspecified)	1 (0.2)	0 (0.0)		
Paired site (no laterality)	0 (0.0)	0 (0.0)		
Bone metastasis			4.45	0.035
Yes	20 (4.1)	8 (1.6)		
No	473 (95.9)	485 (98.4)		
Liver metastasis			30.586	<0.001
Yes	73 (14.8)	21 (4.3)		
No	420 (85.2)	472 (95.7)		
Brain metastasis			28.11	<0.001
Yes	39 (7.9)	4 (0.8)		
No	454 (92.1)	489 (99.2)		
T stage			7.41	0.060
T1	169 (34.3)	142 (28.8)		
T2	194 (39.4)	236 (47.9)		
T3	124 (25.2)	109 (22.1)		
T4	6 (1.2)	6 (1.3)		
N stage			3.05	0.385
N0	198 (40.2)	173 (35.1)		
N1	68 (13.8)	71 (14.4)		
N2	113 (22.9)	130 (26.4)		
N3	114 (23.1)	119 (24.1)		
Tumor size (mm)			0.29	0.593
<67	326 (66.1)	317 (64.3)		
≥67	167 (33.9)	176 (35.7)		
Lymph nodes			5.88	0.118
Regional	3 (0.6)	3 (0.6)		
Distant	253 (51.3)	289 (58.6)		
None	198 (40.2)	173 (35.1)		
Unknown	39 (7.9)	28 (5.7)		
Lymph node size (cm)			3.09	0.378
≤2	67 (13.6)	72 (14.6)		
>2 and ≤5	113 (22.9)	130 (26.4)		
>5	115 (23.3)	118 (23.9)		
None	198 (40.2)	173 (35.1)		

LM, lung metastasis; PSM, propensity score matching.

**Figure 1:**
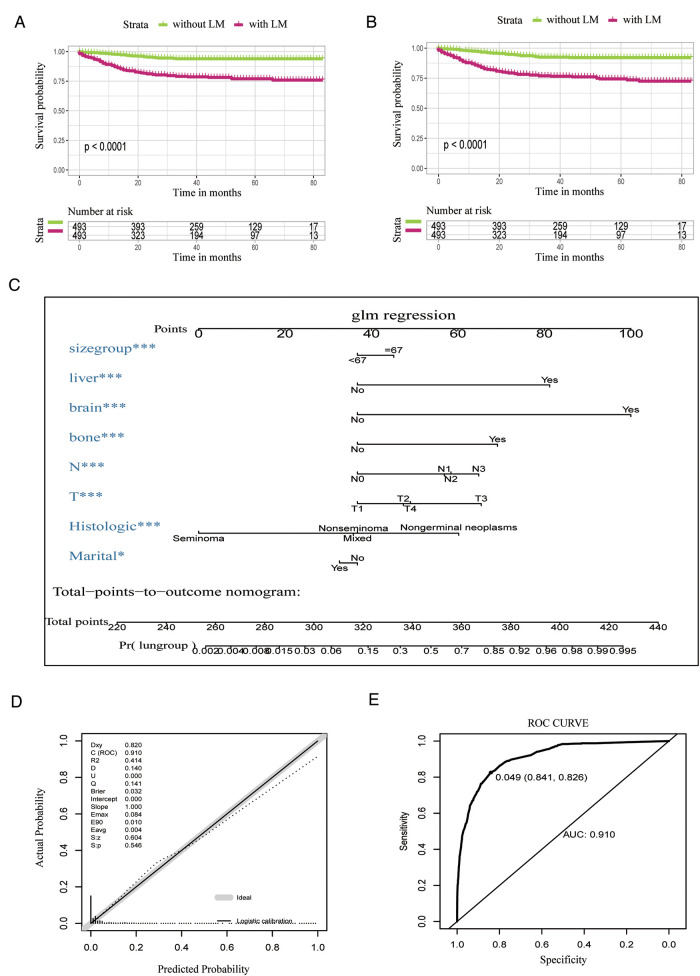
Survival analyses after PSM and the nomogram, the calibration curve for the nomogram, and summarized risks and prognostic factors for TC patients diagnosed with LM. (**A**) OS analysis after PSM. (**B**) CSS analysis after PSM. (**C**) Nomogram predicting the diagnostic possibility for lung metastasis based on the selected variables. (**D**) Logistic calibration curve of the nomogram. (**E**) ROC curve of logistic regression analysis. PSM, propensity score matching; TC, testicular cancer; LM, lung metastasis; OS, overall survival; CSS, cancer-specific survival; ROC, receiver operating characteristic.

Figures 1A,B describe the overall survival (OS) and CSS for 493 cases with LM after PSM, and the curves substantiated that the survival probability of TC patients with LM was significantly lower than those of TC patients without LM in both OS and CSS.

### Risk factors for developing lung metastasis among testicular cancer patients

Univariate logistic regression analysis showed that age [odds ratio (OR) = 0.60, 95% confidence interval (CI): 0.42–0.84, *P* = 0.004], marital status (OR = 0.40, 95% CI: 0.33–0.50, *P* < 0.001), and seminoma (OR = 0.06, 95% CI: 0.04–0.08, *P* < 0.001) were all negatively associated with LM incidence, while tumor grade III (OR = 3.85, 95% CI: 1.84–7.29, *P* < 0.001), nongerminal neoplasm (OR = 3.04, 95% CI: 1.83–4.86, *P* < 0.001), unspecified one-side laterality (OR = 2.02 × 10^0^, 95% CI: 0.80–5.12 × 10^2^, *P* < 0.034), bone metastasis (OR = 32.23, 95% CI: 16.10–66.78, *P* < 0.001), liver metastasis (OR = 74.80, 95% CI: 47.10–123.25, *P* < 0.001), brain metastasis (OR = 212.98, 95% CI: 85.29–712.33, *P* < 0.001), higher T stage (T2/T1: OR = 2.88, 95% CI: 2.33–3.55, *P* < 0.001; T3/T1: OR = 16.25, 95% CI: 12.54–21.02, *P* < 0.001; T4/T1: OR = 6.07, 95% CI: 2.29–13.48, *P* < 0.001), higher N stage (N1/N0: OR = 5.86, 95% CI: 4.36–7.79, *P* < 0.001; N2/N0: OR = 8.53, 95% CI: 6.65–10.89, *P* < 0.001; N3/N0: OR = 12.21, 95% CI: 9.48–15.67, *P* < 0.001), larger tumor size (≥67 mm) (OR = 2.47, 95% CI: 2.03–3.00, *P* < 0.001), and lymph nodes (regional: OR = 3.41, 95% CI: 0.82–9.61, *P* = 0.043; distant: OR = 7.24, 95% CI: 6.01–8.73, *P* < 0.001) were all significantly associated with high risk of LM individually (Table 3).

**Table 3:** Univariate and multivariate logistic regression for analyzing the associated factors for developing lung metastasis in testicular cancer patients.

Subjects’ characteristics	Univariate		Multivariate	
	OR (95% CI)	*P*-value	OR (95% CI)	*P*-value
Age (years)				
<50	1 (reference)	1.00	1 (reference)	1.00
≥50	0.60 (0.42–0.84)	0.004	1.18 (7.49 × 10^−1^–1.79)	0.465
Race				
White	1 (reference)	1.00	1 (reference)	1.00
Black	1.10 (0.63–1.78)	0.709	7.98 × 10^−1^ (4.11 × 10^−1^–1.45)	0.483
Others	1.00 (0.67–1.45)	0.983	9.12 × 10^−1^ (5.51 × 10^−1^–1.45)	0.709
Marital status				
No	1 (reference)	1.00	1 (reference)	1.00
Yes	0.40 (0.33–0.50)	<0.001	7.52 × 10^−1^ (5.84 × 10^−1^–9.63 × 10^−1^)	0.026
Grade				
Unknown	1 (reference)	1.00	1 (reference)	1.00
I	3.54 × 10^−6^ (4.05 × 10^−37^–0.04)	0.954	3.64 × 10^−6^ (2.91 × 10^−52^–3.08)	0.969
II	1.46 (8.04 × 10^−2^–7.28)	0.715	1.47 (7.14 × 10^−2^–9.52)	0.736
III	3.85 (1.84–7.29)	<0.001	8.18 × 10^−1^ (2.93 × 10^−1^–2.04)	0.684
IV	1.86 (5.57 × 10^−1^–4.60)	0.238	9.35 × 10^−1^ (2.56 × 10^−1^–2.64)	0.908
Primary site				
Descended testis	1 (reference)	1.00	1 (reference)	1.00
Testis	0.87 (0.73–1.05)	0.139	1.00 (8.04 × 10^−1^–1.25)	0.988
Undescended testis	0.60 (0.21–1.32)	0.258	7.17 × 10^−1^ (2.07 × 10^−1^–1.92)	0.551
Histology				
Mixed tumor	1 (reference)	1.00	1 (reference)	1.00
Seminoma	0.06 (0.04–0.08)	<0.001	8.05 × 10^−2^ (5.38 × 10^−2^–1.17 × 10^−1^)	<0.001
Nonseminoma	1.00 (0.81–1.25)	0.971	9.36 × 10^−1^ (7.21 × 10^−1^–1.21)	0.616
Nongerminal neoplasm	3.04 (1.83–4.86)	<0.001	4.94 (2.60–8.99)	<0.001
Laterality				
Left	1 (reference)	1.00	1 (reference)	1.00
Right	1.01 (0.84–1.21)	0.946	1.05 (8.43 × 10^−1^–1.30)	0.674
Bilateral	2.59 × 10^−5^ (NA–1.69 × 10^21^)	0.978	5.96 × 10^−6^ (NA–1.04 × 10^106^)	0.994
Only one side (unspecified)	2.02 × 10^0^ (0.80–5.12 × 10^2^)	0.034	2.99 (1.08 × 10^−3^–7.17 × 10^3^)	0.916
Paired site (no laterality)	2.59 × 10^−5^ (NA–8.53 × 10^12^)	0.973	5.25 × 10^−6^ (NA–4.77 × 10^70^)	0.993
Bone metastasis				
No	1 (reference)	1.00	1 (reference)	1.00
Yes	32.23 (16.10–66.78)	<0.001	8.93 (3.51–2.29 × 10^0^)	<0.001
Liver metastasis				
No	1 (reference)	1.00	1 (reference)	1.00
Yes	74.80 (47.10–123.25)	<0.001	2.06 × 10^0^ (1.13 × 10^0^–3.86 × 10^0^)	<0.001
Brain metastasis				
No	1 (reference)	1.00	1 (reference)	1.00
Yes	212.98 (85.29–712.33)	<0.001	7.42 × 10^0^ (2.46 × 10^0^–3.00 × 10^2^)	<0.001
T stage				
T1	1 (reference)	1.00	1 (reference)	1.00
T2	2.88 (2.33–3.55)	<0.001	2.04 (1.60–2.61)	<0.001
T3	16.25 (12.54–21.02)	<0.001	7.02 (5.09–9.67)	<0.001
T4	6.07 (2.29–13.48)	<0.001	2.33 (6.98 × 10^−1^–6.68)	0.138
N stage				
N0	1 (reference)	1.00	1 (reference)	1.00
N1	5.86 (4.36–7.79)	<0.001	4.03 (2.29–6.94)	<0.001
N2	8.53 (6.65–10.89)	<0.001	4.55 (2.65–7.63)	<0.001
N3	12.21 (9.48–15.67)	<0.001	7.15 (4.18–1.19 × 10^0^)	<0.001
Tumor size (mm)				
<67	1 (reference)	1.00	1 (reference)	1.00
≥67	2.47 (2.03–3.00)	<0.001	1.77 (1.37–2.28)	<0.001
Lymph nodes				
None/unknown	1 (reference)	1.00	1 (reference)	1.00
Regional	3.41 (0.82–9.61)	0.043	2.11 × 10^−1^ (3.28 × 10^−2^–8.94 × 10^−1^)	0.060
Distant	7.24 (6.01–8.73)	<0.001	9.76 × 10^−1^ (6.05 × 10^−1^–1.61)	0.923

OR, odd ratio; NA, not available.

Multivariable logistic regression analysis illustrated that marital status (OR = 7.52 × 10^−1^, 95% CI: 5.84 × 10^−1^–9.63 × 10^−1^, *P* = 0.026) is negatively associated with LM. In comparison, nongerminal neoplasm (OR = 4.94, 95% CI: 2.60–8.99, *P* < 0.001), bone metastases (OR = 8.93, 95% CI: 3.51–2.29 × 10^0^, *P* < 0.001), liver metastases (OR = 2.06 × 10^0^, 95% CI: 1.13 × 10^0^–3.86 × 10^0^, *P* < 0.001), brain metastasis (OR = 7.42 × 10^0^, 95% CI: 2.46 × 10^0^–3.00 × 10^2^, *P* < 0.001), higher T stage (T2/T1: OR = 2.04, 95% CI: 1.60–2.61, *P* < 0.001; T3/T1: OR = 7.02, 95% CI: 5.09–9.67, *P* < 0.001), higher N stage (N1/N0: OR = 4.03, 95% CI: 2.29–6.94, *P* < 0.001; N2/N0: OR = 4.55, 95% CI: 2.65–7.63, *P* < 0.001; N3/N0: OR = 7.15, 95% CI: 4.18–1.19 × 10^0^, *P* < 0.001), and larger tumor size (≥67 mm) (OR = 1.77, 95% CI: 1.37–2.28, *P* < 0.001) were all positively associated with LM (Table 3).

Figure 1C (nomogram) illustrates the diagnostic possibility based on the independent risk factors (marital status, tumor size, T stage, N stage, liver, brain and bone metastases, and histologic type). The patient's diagnostic possibility can be calculated by summing the scores for each selected variable, and the total scores projected on the bottom scale represents the diagnostic possibility for LM.

The logistic calibration showed the predicted probability of the nomogram was in line with ideal expectations (Figure 1D). Subsequently, the ROC curve was employed to analyze the validation of the logistic regression model (Figure 1E). The ROC curve showed that the area under the curve (AUC) is 91.0%.

### Cancer-specific survival analysis of testicular cancer patients with lung metastasis

Univariate Cox regression analysis for patients showed that older age (≥50 years) [hazard ratio (HR) = 1.91, 95% CI: 1.02–3.57, *P* = 0.044], tumor grade III (HR = 3.01, 95% CI: 1.22–7.40, *P* = 0.017), bone metastasis (HR = 2.25, 95% CI: 1.09–4.64, *P* = 0.028), brain metastasis (HR = 2.61, 95% CI: 1.50–4.52, *P* = 0.001), liver metastasis (HR = 2.87, 95% CI: 1.84–4.47, *P* = 3.33 × 10^−6^), and larger tumor size (≥67 mm) (HR = 1.53, 95% CI: 1.03–2.29, *P* = 0.037) contributed to the poor prognosis of patients' CSS, while not a single variable was negatively associated with CSS (Table 4).

**Table 4:** Cox regression of univariate and multivariate analyses associated with cancer-specific survival and overall survival of testicular cancer patients with lung metastasis.

Subjects’ characteristics	Cancer-specific survival of testicular cancer patients with LM				Overall survival of testicular cancer patients with LM			
	Univariate		Multivariate		Univariate		Multivariate	
	HR (95% CI)	*P*-value	HR (95% CI)	*P*-value	HR (95% CI)	*P*-value	HR (95% CI)	*P*-value
Age (years)								
<50	1.00 (reference)	1.000	1.00 (reference)	1.000	1.00 (reference)	1.000	1.00 (reference)	1.000
≥50	1.91 (1.02–3.57)	0.044	1.35 (0.66–2.77)	0.416	2.01 (1.13–3.59)	0.018	1.48 (0.77–2.88)	0.242
Race								
White	1.00 (reference)	1.000	1.00 (reference)	1.000	1.00 (reference)	1.000	1.00 (reference)	1.000
Black	0.91 (0.29–2.89)	0.878	0.87 (0.27–2.80)	0.810	1.08 (0.40–2.93)	0.881	1.02 (0.37–2.82)	0.972
Other	1.38 (0.64–2.99)	0.411	1.10 (0.49–2.48)	0.810	1.41 (0.69–2.90)	0.350	1.19 (0.56–2.54)	0.644
Marital status								
No	1.00 (reference)	1.000	1.00 (reference)	1.000	1.00 (reference)	1.000	1.00 (reference)	1.000
Yes	1.20 (0.77–1.88)	0.430	1.39 (0.86–2.27)	0.182	1.78 (0.77–1.80)	0.452	1.36 (0.86–2.16)	0.186
Site								
Descended testis	1.00 (reference)	1.000	1.00 (reference)	1.000	1.00 (reference)	1.000	1.00 (reference)	1.000
Testis	1.05 (0.70–1.56)	0.818	1.11 (0.73–1.68)	0.620	1.03 (0.71–1.49)	0.895	1.09 (0.74–1.61)	0.667
Undescended testis	0.00 (0.00–Inf)	0.994	–	–	0.00 (0.00–Inf)	0.993	–	–
Histology								
Mixed tumor	1.00 (reference)	1.000	1.00 (reference)	1.000	1.00 (reference)	1.000	1.00 (reference)	1.000
Seminoma	1.08 (0.52–2.25)	0.843	1.00 (0.46–2.18)	0.994	1.06 (0.53–2.12)	0.870	0.96 (0.46–2.00)	0.916
Nonseminoma	0.91 (0.57–1.46)	0.693	1.09 (0.66–1.81)	0.725	0.86 (0.55–1.35)	0.514	0.98 (0.60–1.60)	0.945
Nongerminal neoplasm	1.20 (0.48–2.98)	0.700	1.26 (0.47–3.37)	0.646	1.26 (0.55–2.89)	0.591	1.37 (0.56–3.36)	0.495
Grade								
Unknown	1.00 (reference)	1.000	1.00 (reference)	1.000	1.00 (reference)	1.000	1.00 (reference)	1.000
Grade I	–	–	–	–				
Grade II	0.00 (0.00–Inf)	0.996	–	–	5.16 (0,72–37.11)	0.103	5.50 (0.71–42.77)	0,103
Grade III	3.01 (1.22–7.40)	0.017	1.97 (0.73–5.30)	0.181	2.67 (1.09–6.56)	0.032	1.79 (0.67–4.77)	0.247
Grade IV	0.98 (0.14–7.01)	0.981	0.88 (0.11–7.02)	0.907	0.84 (0.12–6.05)	0.865	0.74 (0.09–5.78)	0.773
Laterality								
Left	1.00 (reference)	1.000	1.00 (reference)	1.000	1.00 (reference)	1.000	1.00 (reference)	1.000
Right	1.07 (0.72–1.60)	0.723	1.30 (0.85–1.97)	0.223	1.16 (0.80–1.69)	0.431	1.35 (0.91–2.01)	0.136
Bilateral	–	–	–	–	–	–	–	–
Only one side (unspecified)	0.00 (0.00–Inf)	0.995	–	–	0.00 (0.00–Inf)	0.995	–	–
Paired site (no laterality)	–	–	–	–	–	–	–	–
T stage								
T1	1.00 (reference)	1.000	1.00 (reference)	1.000	1.00 (reference)	1.000	1.00 (reference)	1.000
T2	0.69 (0.44–1.09)	0.110	0.69 (0.43–1.12)	0.132	0.70 (0.46–1.08)	0.109	0.71 (0.45–1.12)	0.143
T3	0.70 (0.41–1.17)	0.170	0.60 (0.34–1.04)	0.066	0.78 (0.48–1.25)	0.298	0.67 (0.40–1.12)	0.126
T4	0.58 (0.08–4.22)	0.590	0.27 (0.03–2.17)	0.219	0.53 (0.07–3.88)	0.535	0.26 (0.03–2.03)	0.199
N stage								
N0	1.00 (reference)	1.000	1.00 (reference)	1.000	1.00 (reference)	1.000	1.00 (reference)	1.000
N1	0.70 (0.36–1.35)	0.283	0.83 (0.32–2.11)	0.690	0.76 (0.41–1.40)	0.373	0.91 (0.38–2.18)	0.826
N2	0.62 (0.36–1.08)	0.093	0.71 (0.30–1.70)	0.449	0.67 (0.40–1.12)	0.129	0.76 (0.33–1.72)	0.506
N3	0.90 (0.55–1.47)	0.663	0.80 (0.35–1.83)	0.602	0.96 (0.60–1.53)	0.865	0.85 (0.39–1.86)	0.688
Bone metastasis								
No	1.00 (reference)	1.000	1.00 (reference)	1.000	1.00 (reference)	1.000	1.00 (reference)	1.000
Yes	2.25 (1.09–4.64)	0.028	1.24 (0.55–2.79)	0.608	1.97 (0.96–4.05)	0.064	1.02 (0.45–2.29)	0.967
Brain metastasis								
No	1.00 (reference)	1.000	1.00 (reference)	1.000	1.00 (reference)	1.000	1.00 (reference)	1.000
Yes	2.61 (1.50–4.52)	0.001	2.01 (1.08–3.73)	0.027	2.62 (1.56–4.39)	0.000	2.11 (1.18–3.78)	0.012
Liver metastasis								
No	1.00 (reference)	1.000	1.00 (reference)	1.000	1.00 (reference)	1.000	1.00 (reference)	1.000
Yes	2.87 (1.84–4.47)	3.33 × 10^−6^	2.52 (1.51–4.22)	<0.001	2.81 (1.85–4.28)	1.43 × 10^−6^	2.52 (1.55–4.10)	<0.001
Tumor size (mm)								
<67	1.00 (reference)	1.000	1.00 (reference)	1.000	1.00 (reference)	1.000	1.00 (reference)	1.000
≥67	1.53 (1.03–2.29)	0.037	1.75 (1.10–2.77)	0.018	1.57 (1.07–2.28)	0.020	1.76 (1.14–2.70)	0.011
Lymph nodes								
None/unknown	1.00 (reference)	1.000	1.00 (reference)	1.000	1.00 (reference)	1.000	1.00 (reference)	1.000
Regional	0.00 (0.00–Inf)	0.994	–	–	0.00 (0.00–Inf)	0.994	–	–
Distant	0.74 (0.49–1.09)	0.132	0.96 (0.45–2.08)	0.926	0.79 (0.55–1.15)	0.224	1.02 (0.49–2.10)	0.961

LM, lung metastasis; HR, hazard ratio.

However, multivariate Cox regression analysis illustrated that brain metastasis (HR = 2.01, 95% CI: 1.56–4.39, *P* < 0.001), liver metastasis (HR = 2.81, 95% CI: 1.85–4.28, *P* = 1.43 × 10^−6^), and larger tumor size (≥67 mm) (HR = 1.57, 95% CI: 1.07–2.28, *P* = 0.020) were all positively associated with the risk of patients' CSS (Table 4).

### Overall survival analysis of testicular patients with lung metastasis

Univariate Cox regression analysis for patients' OS indicated that older age (≥50) (HR = 2.01, 95% CI: 1.13–3.59, *P* = 0.018), tumor grade III (HR = 2.67, 95% CI: 1.09–6.56, *P* = 0.032), brain metastasis (HR = 2.62, 95% CI: 1.56–4.39, *P* < 0.001), liver metastasis (HR = 2.81, 95% CI: 1.85–4.28, *P* = 1.43 × 10^−6^), and larger tumor size (≥67 mm) (HR = 1.57, 95% CI: 1.07–2.28, *P* = 0.020) all positively contributed to patients' OS (Table 4).

However, multivariate Cox regression analysis represented that brain metastasis (HR = 2.11, 95% CI: 1.18–3.78, *P* = 0.012), liver metastasis (HR = 2.52, 95% CI: 1.55–4.10, *P* < 0.001), and larger tumor size (≥67 mm) (HR = 1.76, 95% CI: 1.14–2.70, *P* = 0.011) all were positively associated with the risk of patients' OS (Table 4).

## Discussion

In recent decades, TC has become the most prevalent solid cancer in men aged 14–44 years, and the international trends of incidence have been increasing (1, 15). Poor outcomes in TC are driven primarily by metastatic involvement (clinical stage III disease). Among patients with tumor metastasis (lung, lymph node, liver, and central nervous system), predictors of survival are multifactorial (6, 16). Given the development of multimodal strategies to manage lung metastatic TC, patients tend to exhibit improved clinical outcomes. However, the relative risk factors that removed confounding parameters on survival among TC patients with LM have not been systematically evaluated. Herein, we systematically assess the impact of the LM on survival using a large, nationally population-based cancer cohort. Clarifying the specific influence of organotropism on survival outcomes might improve current prognostic models for lung metastatic TC and provide insight into the heterogeneity of TC.

For lung metastasis TC, platinum-based chemotherapy is typically utilized as an adjuvant treatment after radical orchiectomy combined with multimodality treatments. Adjuvant chemotherapy with platinum/etoposide/bleomycin (BEP) is the commonly used chemotherapy regimen, which dramatically improves the outcome for patients with metastatic TC (17). However, chemotherapy for metastatic TC remains incurable and the poor prognosis now exceeds 20% (18). Therefore, radiotherapy or surgical management is developed for adjunctive therapy to chemotherapy. Studies revealed a promising improvement in OS of thoracic metastasectomy/pulmonary resection (19, 20). To achieve an optimal curative effect, postchemotherapy surgery retroperitoneal lymph node dissection is a critical procedure of metastatic TC management (21). PSM is a prevalent analytic method that may remove the effects of confounding biases due to measured baseline covariates when estimating the outcomes using observational data (14). PSM was employed in the analysis for CSS and OS to abbreviate the confounding biases of several variates and evaluate whether LM is an independent risk factor for survival probability in TC patients. After PSM, OS, and CSS between 493 groups were analyzed, and a higher survival probability was observed among patients without LM compared to those with LM. The application of the PSM method definitely increases the reliability and accuracy of our model.

For the first time, we utilized the largest real-world SEER database (2010–2015) to determine the related risk factors for TC patients with LM. Our research based on the logistic regression model revealed that nongerminal neoplasm, bone metastasis, liver metastasis, brain metastasis, higher T stage, higher N stage, and larger tumor size are independent risk factors positively related to LM development; marital status is a protective factor in TC patients with LM. Therefore, we suggest that the LM risk of TC patients should be carefully revalued in the contemporary cohort according to these risk factors. In this study, we also found that a higher T stage and higher N stage were associated with a greater possibility of LM, which commonly illustrates more aggressive biological behavior of cancer and worse physical status. In addition, previous studies demonstrated the importance of racial disparities in developing TC in young-onset patients (22, 23). However, race is not a risk factor for developing TC with LM in our study (*P-*value = 0.933).

A nomogram was constructed using our logistic regression model to demonstrate the diagnostic possibility based on the independent risk variates (marital status; tumor size; T stage; N stage; liver, brain, and bone metastases; and histologic type). The diagnostic possibility can be estimated by adding the score of each selected variate, and the total scores projected on the bottom scales presents the diagnostic possibility for LM. Nomograms allow medical practitioners to assess the patients' physical situation more accurately and intuitively to evaluate the personalized prediction for cancer prognosis. Subsequently, we applied the ROC curves to verify the predictive capacity of the prognostic model; the AUCs of the ROC were 0.910, indicating a favorable predictive performance. Mao et al. and Wu et al. reported nomograms for predicting survival in germ cell TC, which showed a moderate diagnostic value (0.7–0.9) (23).

The Cox regression model was utilized to determine the predictive factors for CSS and OS among TC patients with LM based on the SEER database. Brain and liver metastases and larger tumor size (≥67 mm) were observed to be positively associated with the risk of patients' CSS and OS in multivariate analysis. Notably, our study is the first to address the role of tumor size in lung metastatic TC patients' survival using a contemporary large-sample cohort. Interestingly, a previous study revealed that testicular tumor size is associated with relapse (24). Furthermore, our study is consistent with previous studies (5, 6) that patients with involvement of liver or brain metastatic sites presented worse outcomes than those with bone metastasis. The tendency of a tumor to metastasize to particular organ sites might reflect an interplay between the underlying biology of the tumor cells and a permissive host organ microenvironment (25–27).

At last, despite the advantages of our study, there exist some limitations. First, potential biases among children, adolescents, and young adults exist in our study, which may affect our results to some extent. Second, detailed information for diagnosing LM is unavailable in this SEER database, thus adding much uncertainty to the current amount of TC patients with LM. In addition, the lack of external validation due to the low number of cases in other cohorts is a limitation of our study. Given the retrospective nature, there are clinical parameters associated with the treatments that were not included or missed. Finally, subanalyses could be performed between young and older TC patients with LM to get a comprehensive pattern of TC.

## Data Availability

The original contributions presented in the study are included in the article/Supplementary Material; further inquiries can be directed to the corresponding author/s.
